# Cerebral amyloid angiopathy-related acute lobar intra-cerebral hemorrhage: diagnostic value of plain CT

**DOI:** 10.1007/s00415-021-10796-z

**Published:** 2021-09-12

**Authors:** Jean-Claude Baron, Grégoire Boulouis, Joseph Benzakoun, Corentin Schwall, Catherine Oppenheim, Guillaume Turc, Pascale Varlet

**Affiliations:** 1 Department of Neurology, Groupe Hospitalier Universitaire de Paris, Université de Paris, Paris, France; 2 Inserm U1266, Institut de Neurosciences et de Psychiatrie de Paris, Paris, France; 3 Department of Radiology, CHU de Tours, Tours, France; 4 Department of Imaging, Groupe Hospitalier Universitaire de Paris, Université de Paris, Paris, France; 5 Department of Pathology, Groupe Hospitalier Universitaire de Paris, Université de Paris, Paris, France

**Keywords:** Boston criteria, MRI, Pathology, Subarachnoid hemorrhage, Finger-like projections

## Abstract

**Background:**

Diagnosing probable cerebral amyloid angiopathy (CAA) after lobar intra-cerebral hemorrhage (l-ICH) currently relies on the MR-based modified Boston criteria (mBC). However, MRI has limited availability and the mBC have moderate sensitivity, with isolated l-ICH being classified as “possible CAA”. A recent autopsy-based study reported potential value of finger-like projections (FLP) and subarachnoid hemorrhage (SAH) on acute CT. Here we assessed these markers’ performance in a cohort most of whom survived the index episode.

**Methods:**

We included all patients from a prospective pathology database with non-traumatic l-ICH, admission CT and available tissue sample showing no alternative cause. CT was assessed by two blinded independent neuroradiologists. Interobserver reproducibility was almost perfect for SAH and substantial for FLP.

**Results:**

Sixteen patients were eligible [age 65.8 ± 7.2 yrs; hematoma volume: 39(26, 71)mls; hematoma evacuation sample 15 patients; autopsy one patient]. MRI was available in 11 patients. ICH-related death affected six patients. Aβ_40–42_ immunohistochemistry revealed CAA in seven patients (44%). SAH and FLP were present in 12/16 (75%) and 10/16 (62%) patients, respectively. SAH had 100% sensitivity for CAA but low specificity; FLP had lower performance. Using either pathology or MRI as reference standard yielded essentially similar results. All patients with possible CAA on MRI but CAA on pathology had SAH.

**Conclusions:**

In patients with moderate-size l-ICH who mostly survived the index event, SAH had perfect sensitivity and better performance than FLP. In addition, SAH appeared to add onto MRI in possible CAA, the clinically most relevant scenario. Studies in larger samples are however warranted.

## Introduction

The modified Boston criteria for the diagnosis of probable CAA are based on MRI hemorrhagic markers exclusively involving lobar regions [1, 2]. Making a diagnosis of CAA may have important consequences for both prognostication and patient care, notably regarding management of blood pressure and use of anti-thrombotic agents, but also in the context of patient selection for research studies and drug trials. However, MRI may not be available in all clinical settings worldwide, particularly in the hyper-acute setting, whereas CT is recommended routine first-line diagnostic imaging in patients with acute stroke. To address the question whether plain CT has diagnostic value for CAA, Rodrigues et al. published the results of a prospective study of patients with intra-cerebral hemorrhage (ICH) who underwent CT as part of initial diagnostic work-up, who subsequently died (median delay 11 days), and in whom full research autopsy was available as part of a prospective population-based registry [3]. Focusing on lobar ICH (*N* = 62 patients; CAA present pathologically in 58%), they assessed the diagnostic value of two potential hemorrhagic CT markers, the finger-like projections (FLP) of the hematoma and the presence of acute sub-arachnoid hemorrhage (SAH) near the hematoma, and one biological marker, the APO-E genotype. Although combination of all three markers had an excellent positive predictive value (PPV; 96%), APO-E genotyping is not part of clinical practice, and in many countries formal consent is required to carry out genotyping, as with any genetic study. Taken separately, the diagnostic value of these two CT markers was as follows: (i) FLP showed perfect (100%) specificity, i.e., no false positive, but weak sensitivity (39%), indicating that many patients with autopsy-proven CAA did not have FLP; and (ii) conversely, SAH was present in 89% of the proven CAA cases (sensitivity) but also in 42% of the non-CAA cases, for a moderate specificity of 58%.

These novel findings not only open up new avenues in the acute diagnosis of CAA presenting with inaugural ICH, but may also improve the sensitivity of the MR-based Boston criteria and allow diagnosing CAA even after isolated lobar hemorrhage, i.e., “possible CAA”. In a subsequent study of lobar ICH in hereditary Dutch-type CAA, van Etten et al. [4] reported a sensitivity of 58% and 76% for FLP and SAH, respectively, which however markedly declined with smaller hematoma size. Because by definition all their subjects had hereditary CAA, they were unable to assess specificity.

In the present single-center pilot study, we aimed to test the diagnostic value of FLP and SAH on admission CT in a cohort of symptomatic lobar ICH patients in whom pathological diagnosis was available mostly as hematoma evacuation sample. Furthermore, most patients survived the index event, as is true for the majority (≈57%) of the symptomatic lobar ICH population [5]. This cohort can be therefore considered paradigmatic of survivors from lobar ICH presentation in whom making a diagnosis of CAA has particular relevance.

## Methods

The prospective database of the Pathology Department of GHU-Paris, Sainte-Anne site, University of Paris, was screened (1999–2019), and all cases under the category “hemorrhage” were retrieved. Were eligible for the present study all consecutive patients with (i) non traumatic lobar intra-parenchymal hemorrhage; (ii) plain CT obtained on patient admission; (iii) available histological analysis of either hematoma neurosurgical evacuation with samples containing at least brain and/or leptomeningeal arterioles, or autopsy material, allowing to search for vascular amyloid as clinical diagnostic routine [6]; and (iv) who did not have a final diagnosis of vascular malformation, tumor or any other local process as underlying cause for the hematoma. For each eligible patient the demographics and baseline clinical characteristics were retrieved from the medical files.

As for all non-interventional retrospective studies of anonymised data in France, written informed consent was waived. A commitment to compliance regarding the use of these data was filed to the National Information Science and Liberties Commission (CNIL) prior to data centralization, in agreement with the General Data Protection Regulation.

### Neuropathological assessment

The brain hematomas were neurosurgically evacuated using standard procedures and consistently fixed in formaldehyde/zinc, again using standard clinical procedures. The small parts of brain tissue attached to the hematoma were carefully assessed neuropathologically and processed for Aβ_40-42_ immuno-histochemistry, consistently using the same technique to ensure staining homogeneity.

The tissue samples were assessed by an experienced neuropathologist (PV) blinded to the radiological and clinical data. The presence or absence of Aβ_40–42_ deposits within the wall of arterioles was assessed. Their severity was rated according to Vonsattel et al. [7, 8], as absent, mild (i.e., focal amyloid deposit; Vonsattel Grade (1), moderate (circumferential amyloid deposit; Vonsattel Grade (2) or severe (circumferential with double-lumen or vessel obstruction; Vonsattel grades 3–4). A diagnosis of CAA was made based on Vonsattel Grade ≥ 1 for evacuation samples [6, 7], or Vonsattel Grades 2–4 for autopsy material [6–8].

### Radiological assessment

The acute-stage admission non-contrast enhanced CT (NCCT) for each patient was assessed by two independent experienced neuroradiologists (GB, JB) blinded to clinical, neuropathological and any other radiological data, for (i) FLP; and (ii) SAH, using the definitions and methodology described in Rodrigues et al. [3], after validating the recommended training using the Edinburgh Criteria for CAA-associated ICH Training (ECCITING) available at https://www.ed.ac.uk/clinical-sciences/edinburgh-imaging/education-teaching/short-courses/training-tools/edinburgh-criteria-for-caa-associated-ich-training. Inter-observer reproducibility was calculated for FLP and SAH separately, using Cohen’s Kappa. Any discrepancy was resolved by consensus.

The ICH volume was calculated using the ellipsoid approximation as ABC/2 or ABC/3 for irregular or multinodular hemorrhages, as previously reported [4].

In addition, whenever available, MRI datasets including GRE/T2* sequences obtained as part of clinical routine closest in time to the index hemorrhage were assessed by an experienced neuroradiologist (GB), blinded to all other data, for the presence of hemorrhagic markers of CAA including cerebral microbleeds, cortical superficial siderosis and prior hemorrhages according to the standard reporting methods developed by the STandards for ReportIng Vascular changes on nEuroimaging (STRIVE) consensus [9].

### Statistical analysis

In the main analysis, the pathological diagnosis of CAA was considered the gold standard. The presence of FLP, SAH, either FLP or SAH, or both FLP and SAH was compared to the gold standard, and true positives, false positives, true negatives and false negatives were determined, allowing in turn the sensitivity, specificity, positive predictive value (PPV), negative predictive value (NPV) and accuracy to be computed.

In the secondary analysis, the same calculations were made except that we considered as reference standard a diagnosis of CAA based on either pathology or MRI, whenever available. The rationale behind this is that hematoma evacuation might be negative despite clinically likely CAA [10], whereas the MR-based modified Boston Criteria have excellent specificity but only moderate sensitivity [2, 6, 11].

In an ancillary analysis, we asked whether, using pathology as gold standard, CT markers improve upon the diagnostic performance of MRI, more specifically its sensitivity. In other words, we assessed whether CT markers were present in situations where an MRI-based diagnosis of probable CAA could not be made despite pathology showing CAA. Because per protocol all our patients had lobar hematoma, this meant actual reclassification of modified Boston criteria-based diagnosis of “possible” into “probable” CAA.

## Results

### Clinical material

Sixteen patients with complete dataset were available. Key demographics and clinical data are presented in Table 1. Median [IQR] hematoma volume was 39 [26, 71] mL. Pathological diagnosis was based on hematoma evacuation in 15/16 patients. Early ICH-related death occurred in six patients (all < 6 months after stroke onset). MRI was available in 11 patients and missing in five patients, mostly because of early death precluding performance of an MRI.

**Table 1:** Demographics and main lobar hematoma-related variables

Patient #	Age/sex	Year*	Vascular risk factors	Antithrombotics	Side/location	Volume (mls)	Pathological material	MRI	Lobar ICH-related death
1	55/F	2015	HTN	No	L/frontal	27	HE	Yes	Yes
2	74/F	2017	HTN	No	L/occipital	32	HE	No	No
3	57/F	2017	HTN, NIDM	Heparin	R/fronto-parietal	38	HE	Yes	Yes
4	80/F	2018	HTN, DL	No	R/frontal	8	HE	Yes	No
5	76/F	2019	None	No	L/parietal	40	HE	Yes	No
6	67/F	2019	HTN	No	Parietal	35	HE	Yes	No
7	71/F	2019	None	No	L/frontal	89	HE	Yes	Yes
8	69/F	2019	HTN	No	R/temporal	40	HE	Yes	No
9	59/M	2010	HTN	No	L/temporo-occipital	20	HE	Yes	No
10	65/M	2013	DL	Aspirin	R/frontal	82	HE	Yes	No
11	60/M	2013	HTN, NIDM	Aspirin	Frontal	81	HE	No	Yes
12	67/F	1999	None	No	Frontal	26	HE	No	No
13	63/F	2004	None	Aspirin	Frontal	15	Autopsy	No	Yes
14	69/M	1999	NIDM	No	Frontal	60	HE	Yes	No
15	62/M	1999	DL	No	Frontal	74	HE	No	Yes
16	59/M	2006	HTN, smoking	No	Frontal	45	HE	Yes	No

^*^: year the pathological material was available; *F* female, *M* male, *HTN* arterial hypertension, *NIDM* non-insulin dependent diabetes mellitus, *DL* dyslipidemia, *L* left, *R* right, *HE* hematoma evacuation

Pathological diagnosis, occurrence of FLP and SAH, and diagnosis based on MRI are shown in Table 2. A pathological diagnosis of CAA was made in 7/16 (44%) patients. Interobserver reproducibility was almost perfect for SAH (Kappa = 0.88; 95% CI 0.65–1.00) and substantial for FLP (Kappa = 0.67; 95% CI 0.36–0.99). Based on the modified Boston criteria, an MRI-based diagnosis of probable CAA could be made in 3/11 patients.

**Table 2:** Presence of subarachnoid hemorrhage (SAH) and finger-like projection (FLP) on admission CT, and CAA diagnosis as per pathological findings, MRI markers (modified Boston criteria), or either pathology or MRI

Patient	SAH	FLP	Pathology	MRI	Pathology or MRI
1	1	1	0	Yes	1
2	1	1	1 (severe)	No	1
3	0	1	0	NA	0
4	0	0	0	No	0
5	1	1	1 (moderate)	No	1
6	1	0	0	Yes	1
7	1	1	1 (moderate)	Yes	1
8	1	0	0	No	0
9	0	0	0	No	0
10	0	1	0	No	0
11	1	1	0	NA	0
12	1	1	1 (moderate)	NA	1
13	1	1	1 (severe)	NA	1
14	1	0	1 (severe)	No	1
15	1	0	1 (mild)	NA	1
16	1	1	0	No	0

*1* present, *0* absent, *NA* not available

### Diagnostic performance of CT hemorrhagic markers

The performance of the two hemorrhagic CT markers is presented in Table 3.

**Table 3:** Specificity, sensitivity, PPV and NPV of CT-based markers (FLP and SAH) against CAA diagnosis according to pathology (A; *n* = 16), MRI (B; *N* = 11) and Pathology or MRI (C; *n* = 16; of which 11 had MRI)

	Specificity	Sensitivity	PPV	NPV	Accuracy
A					
FLP	44%	71%	50%	67%	56%
SAH	44%	100%	58%	100%	69%
FLP or SAH	22%	100%	50%	100%	56%
FLP and SAH	67%	71%	63%	75%	69%
B					
FLP	50%	67%	33%	80%	55%
SAH	38%	100%	38%	100%	55%
C					
FLP	43%	67%	60%	50%	56%
SAH	57%	100%	75%	100%	81%
FLP or SAH	29%	100%	64%	100%	69%
FLP and SAH	71%	67%	75%	63%	69%

In the *main analysis* (Table 3A), FLP showed poor specificity (44%) and moderate sensitivity (71%), while SAH had perfect (100%) sensitivity and NPV (i.e., when SAH was absent, the pathological diagnosis was not CAA), but poor/moderate specificity and PPV (44% and 58%, respectively), which indicates that when SAH was present, the pathological diagnosis may or may not be CAA. The overall accuracy of SAH was 69%. Using the presence of either FLP or SAH, sensitivity remained at 100% as expected, but specificity further deteriorated. Using the presence of both FLP and SAH (occurrence 8/16 patients) increased specificity but markedly decreased sensitivity. From these results, it would appear that SAH has greater clinical relevance than FLP, and using both has no clear added value.

Using MRI as reference standard instead of pathology, diagnostic performance of the CT markers (Table 3B) was quite similar to that using pathology, ie moderate/poor performance of FLP, and still 100% sensitivity but poor specificity of SAH.

In the *secondary analysis*, using either pathology or MRI as reference standard (Table 3C) yielded results very similar to those obtained using pathology, except that the accuracy of SAH increased to 81%, while using the presence of both FLP and SAH resulted in reasonable specificity, sensitivity, PPV, NPV (63–75%) but with lower accuracy (69%) than with SAH alone.

In the ancillary analysis, carried out in the subset of 11 patients in whom MRI was available, MRI did not allow to make a diagnosis of probable CAA in three patients with a pathological diagnosis of CAA. SAH and FLP were present in all three, and in two patients, respectively.

## Discussion

Following the publication of the autopsy-based Edinburgh criteria [3], our aim here was to assess the diagnostic value of FLP and SAH for CAA in a cohort of symptomatic lobar ICH patients most of whom survived the acute episode. A further notable difference from this previous study was that here the pathological material almost exclusively consisted of hematoma evacuation.

In the present study, the pathological diagnosis of CAA was based on recommended criteria, namely Vonsattel Grade ≥ 1 for evacuation samples, or Vonsattel Grades ≥ 2 for autopsy material [6–8]. In Greenberg and Vonsattel’s seminal study [7], the presence of Vonsattel Grade 1 on cortical samples had 100% sensitivity to detect definite CAA as documented on whole brain assessment. The specificity declined with advancing age but remained > 95% until age 75 yrs [7]. Because only two patients in our cohort were older than this, this issue likely has limited impact on our findings.

One striking finding from our study is the high prevalence of both FLP and SAH in our cohort of lobar ICH, present in 12/16 (75%) and 10/16 (62%) patients, respectively. Prevalence of SAH in our cohort is similar to that reported by both Rodrigues et al. [3] in mixed causes ICH and van Etten et al. [4] in CAA-only lobar ICH (69% and 76%, respectively), and prevalence of FLP is similar to that found in the latter study (58%). However, Rodrigues et al. [3] reported a considerably lower prevalence of FLP, namely 23%. The reason for this discrepancy with our and van Etten et al.’s study is unclear. Our raters were senior neuroradiologists who both validated trained using Edinburgh’s dedicated online system (see Methods), and inter-rater reproducibility was higher than in Rodrigues et al.’s study [3] (kappa = 0.88 and 0.67 for SAH and FLP in our study, vs 0.71 and 0.60, respectively). Although in our study the final rating was made by consensus, i.e., the conventional approach, for unexplained reasons Rodrigues et al. used the ratings by one of their raters only [3]. ICH volume differences are unlikely to explain this discrepancy. Both van Etten et al. [4] and more recently Ornello et al. [12] have reported a strong positive association between the presence of both CT markers and lobar ICH volume. Yet, as expected given the differences in cohort characteristics, hematoma volume was much smaller in our study (median 39 mL) as compared to Rodrigues et al. [3] (median value for lobar hematomas not provided but certainly > 60 mL based on their Table 1). A recent study [13] where pathological diagnosis was not available reported a low prevalence of FLP (37%) in association with low lobar ICH volume (median 27 mL); however, CT was not consistently obtained in the acute stage in this study. In Ornello et al. [12], the prevalence of SAH and FLP was 63% and 30%, respectively, but lobar hematoma volume is not reported.

In our study either marker was present in 14/16 patients (87%) and both in 8/16 (50%), and 2/16 (6%) patients only had FLP alone. These numbers are similar to van Etten’s study [4] in subjects with hereditary CAA-associated lobar ICH (82%, 52% and 6%, respectively). In Ornello et al. [12], 64% had either marker and 0.6% isolated FLP; however, it is unclear at which time point was NCCT obtained. Taken together, these observations indicate that the vast majority of patients with lobar ICH have at least one CT hemorrhagic marker, while isolated FLP is a rare finding. The latter observation, added onto the limited inter-rater reproducibility of FLP assessment, suggests that SAH may be a more relevant CT marker for CAA diagnosis than FLP.

In the primary analysis, we found that the presence of FLP had moderate sensitivity and poor specificity regardless of the reference standard—namely, pathology, MRI or both—, whereas, SAH had perfect sensitivity and NPV—i.e., when SAH is absent the diagnosis of CAA can be safely ruled out—, but moderate specificity (50–67%)—i.e., its presence does not really help with the etiological diagnosis. These findings may suggest potential face-value utility of plain CT in clinical practice to rule out, but not to rule in, CAA.

These results substantially differ from those reported by Rodrigues et al. [3], who found that FLP had perfect specificity, i.e., no false positives, but weak sensitivity (39%), i.e., many patients with autopsy-proven CAA did not have FLP, whereas, conversely SAH had good sensitivity (89%) but moderate specificity (58%). Apart from the smaller sample here (*n* = 16 *vs* *n* = 62 patients), reasons for this discrepancy may include smaller hematomas allowing survival in the majority of patients (10/16), and use of mostly hematoma evacuation samples (15/16) as compared to autopsies only in Rodrigues et al. [3] The single patient with autopsy from the present series had both FLP and SAH and showed CAA on pathology, fitting well with Rodrigues’s findings. Excluding this patient *post-hoc* did not substantially change our findings, however (data not shown). van Etten et al. [4] reported sensitivities of 58% and 76% for FLP and SAH, respectively, values that are quite similar to our results; because by definition all their patients had CAA, specificity could not however be assessed. Hematoma volume was smaller in the latter study (median 16mls) than here, and the authors reported higher sensitivity of both SAH and FLP with larger volumes.

Although as indicated above the sensitivity of SAH was perfect in our study, its specificity was moderate. Considering the presence of both FLP and SAH together improved specificity and NPV to potentially useful clinical range, namely ~ 70% for both, although at the cost of reduced sensitivity (also ~ 70%). Unfortunately, the diagnostic performance of the presence of both SAH and FLP is not reported in Rodrigues et al. [3]. In van Etten’s study [4], the sensitivity of the presence of both SAH and FLP was highly dependent on hematoma volume, namely ~ 80% vs < 50% for volumes > 40mls and < 15 mL, respectively. In our study, only two hematomas were ≤ 15 mL (Table 1), and excluding them *post-hoc* did not significantly change the sensitivity/specificity values (data not shown). Because the absence of SAH alone ruled out CAA in our study, whether absence of both SAH and FLP has clinical utility is irrelevant.

As pointed out above, we considered CAA to be present on hematoma evacuation even if of mild severity. Rodrigues et al., using autopsy data, considered only moderate/severe CAA as diagnostic [3], as recommended [7]. Excluding *post-hoc* the single patient with mild CAA from our analysis did not alter the results (data not shown).

What is the diagnostic value of FLP and SAH for patients who survive their hemorrhagic stroke? This is a clinically relevant question because the data reported by Rodrigues et al. [3] concern only patients who died from their stroke. Excluding the six patients with ICH-related death in our sample did not substantially change the results, specifically SAH still showed 100% sensitivity regardless of the reference standard used, still with low specificity (50–60%), while FLP’s sensitivity and specificity stand both around 70%. These values have however to be taken with caution given the very small sample (*N* = 10).

The above discussion focused on the potential diagnostic value of admission NCCT in lobar ICH. However, in current practice—be it for routine or research purposes—to make a diagnosis of CAA *vs* no-CAA in an ICH patient is not urgent given that early management is independent of the underlying cause. The diagnostic issue usually arises at a later stage, when decisions need to be made regarding notably starting/re-starting antithrombotics or not [14]. In this scenario, there is generally ample time to carry out an MRI with gradient-echo sequences to assess the modified Boston criteria. As already mentioned, MRI has excellent specificity but low sensitivity, as illustrated by the daily clinical situation of symptomatic single lobar ICH without any additional hemorrhagic marker on MRI, i.e. “possible CAA”. Accordingly, a clinically relevant issue is whether CT adds on to the diagnostic value of MRI. To address this question we assessed whether either CT marker was present whenever MRI did not meet the criteria for probable CAA yet pathology showed CAA. Although our results are clearly limited by the small sample with available MRI, it is of interest to note that SAH was present in all three such patients (and FLP in two). Thus, CT allowed accurate reclassification of all three cases with an MR-based diagnosis of “possible CAA”. Were this observation to be confirmed in larger samples, this would represent relevant clinical utility of admission NCCT in lobar ICH.

Of note, two patients with negative hematoma evacuation had a diagnosis of probable CAA on MRI, pointing to a possibly limited sensitivity of hematoma evacuation for CAA [10].

In addition to the small sample limiting robustness and precluding formal statistical comparisons, this pilot study has all the limitations of a single-center, retrospective design, including potential selection bias. However, all consecutive cases in our pathology laboratory intracranial hemorrhage prospective registry were assessed, reflecting daily practice. All our patients but one underwent acute surgical hematoma evacuation, which may constitute a selection bias and limit generalizability of our observations. One autopsy case only was available because the rate of autopsies has dramatically declined in recent decades in France. Only 11/16 patients had an MRI, which was missing mainly because of ICH-related death.

## Conclusions

Although limited by the small sample and retrospective design, this study suggests that SAH may be a novel useful diagnostic criterion for CAA, allowing to rule out CAA if absent. In addition, SAH may add to the sensitivity of the MRI-based Boston criteria in case of isolated lobar ICH without other hemorrhagic CAA markers, i.e., possible CAA. Further studies in larger samples are needed to explore these promising avenues.

## Data Availability

The data are available from the corresponding author upon reasonable request.

## Code availability

N/A.
